# Whole-genome sequencing of a quarter-century melioidosis outbreak in temperate Australia uncovers a region of low-prevalence endemicity

**DOI:** 10.1099/mgen.0.000067

**Published:** 2016-07-11

**Authors:** Stephanie N. J. Chapple, Derek S. Sarovich, Matthew T. G. Holden, Sharon J. Peacock, Nicky Buller, Clayton Golledge, Mark Mayo, Bart J. Currie, Erin P. Price

**Affiliations:** ^1^​ Melbourne Medical School, University of Melbourne, Melbourne, Victoria, Australia; ^2^​ Global and Tropical Health Division, Menzies School of Health Research, Darwin, Northern Territory, Australia; ^3^​ School of Medicine, Medical and Biological Sciences, University of St Andrews, St Andrews, UK; ^4^​ Wellcome Trust Sanger Institute, University of Cambridge, Cambridge, UK; ^5^​ University of Cambridge, Cambridge, UK; ^6^​ Department of Agriculture and Food Western Australia, Perth, Western Australia, Australia; ^7^​ Sir Charles Gairdner Hospital, Perth, Western Australia, Australia; ^8^​ Department of Infectious Diseases and Northern Territory Medical Program, Royal Darwin Hospital, Darwin, Northern Territory, Australia

**Keywords:** melioidosis, *Burkholderia pseudomallei*, evolution, endemicity, Australia, temperate

## Abstract

Melioidosis, caused by the highly recombinogenic bacterium *Burkholderia pseudomallei*, is a disease with high mortality. Tracing the origin of melioidosis outbreaks and understanding how the bacterium spreads and persists in the environment are essential to protecting public and veterinary health and reducing mortality associated with outbreaks. We used whole-genome sequencing to compare isolates from a historical quarter-century outbreak that occurred between 1966 and 1991 in the Avon Valley, Western Australia, a region far outside the known range of *B. pseudomallei* endemicity. All Avon Valley outbreak isolates shared the same multilocus sequence type (ST-284), which has not been identified outside this region. We found substantial genetic diversity among isolates based on a comparison of genome-wide variants, with no clear correlation between genotypes and temporal, geographical or source data. We observed little evidence of recombination in the outbreak strains, indicating that genetic diversity among these isolates has primarily accrued by mutation. Phylogenomic analysis demonstrated that the isolates confidently grouped within the Australian *B. pseudomallei* clade, thereby ruling out introduction from a melioidosis-endemic region outside Australia. Collectively, our results point to *B. pseudomallei* ST-284 being present in the Avon Valley for longer than previously recognized, with its persistence and genomic diversity suggesting long-term, low-prevalence endemicity in this temperate region. Our findings provide a concerning demonstration of the potential for environmental persistence of *B. pseudomallei* far outside the conventional endemic regions. An expected increase in extreme weather events may reactivate latent *B. pseudomallei* populations in this region.

## Data Summary

1. All Illumina data from this study were uploaded to public databases. MSHRs 0161, 0169 and 0173 were deposited in the European Nucleotide Archive database and are available at http://www.ebi.ac.uk/ena/data/view/ERS205900, http://www.ebi.ac.uk/ena/data/view/ERS205902 and http://www.ebi.ac.uk/ena/data/view/ERS205903, respectively.
2. MSHRs 0157, 0160, 0162, 0163, 0167, 0170, 0171 and 0172 were deposited in the NCBI Sequence Read Archive database and are available at http://trace.ncbi.nlm.nih.gov/Traces/sra/?run=SRR2134233, http://trace.ncbi.nlm.nih.gov/Traces/sra/?run=SRR2134234, http://trace.ncbi.nlm.nih.gov/Traces/sra/?run=SRR2134235, http://trace.ncbi.nlm.nih.gov/Traces/sra/? run=SRR 2 1 3 4 2 3 7 , http://trace.ncbi.nlm.nih.gov/Traces/sra/?run=SRR2134238, http://trace.ncbi.nlm.nih.gov/Traces/sra/?run=SRR2134239, http://trace.ncbi.nlm.nih.gov/Traces/sra/?run=SRR2134240 and http://trace.ncbi.nlm.nih.gov/Traces/sra/?run=SRR2134242, respectively.
3. The MSHR0169 assembly has been deposited in GenBank under accession number LGKL00000000 (http://www.ncbi.nlm.nih.gov/nuccore/LGKL00000000).

## Impact Statement

Melioidosis, a potentially deadly infectious disease caused by the bacterium *Burkholderia pseudomallei*, has conventionally been considered a disease confined to subtropical and tropical regions. There is mounting evidence that *B. pseudomallei* can survive and persist long-term in soil and water in temperate regions that are well outside established regions of melioidosis endemicity. However, it is not yet understood whether the bacterium is being sporadically introduced from endemic tropical regions following severe weather events, or whether it is in fact endemic in these temperate regions, remaining quiescent for years or even decades until its reactivation following unusually heavy rains and flooding. We used whole-genome sequencing to compare isolates from an historical, quarter-century outbreak that occurred between 1966 and 1991 in the temperate Avon Valley, a region of farmland, National Park and scrub, 60 km north-east of Perth, Western Australia. Our whole-genome sequencing-based analysis suggests, for the first time, the long-term, low-prevalence endemicity of *B. pseudomallei* in this temperate region. An increase in severe weather events as a result of climate change may lead to a spike in melioidosis cases being reported in temperate regions globally, and should be factored into future melioidosis modelling scenarios.

## Introduction

Melioidosis is an emerging tropical infectious disease with high mortality that is caused by the Gram-negative bacterial saprophyte *Burkholderia pseudomallei*. The *B. pseudomallei* genome is more complex than most bacterial genomes: not only is it large at 7.2 Mbp, it is subject to high rates of lateral gene transfer (Pearson *et al.*, 2009) and possesses several regions of low complexity, as evidenced by a high G+C content (69 %) and a large cache of repetitive loci (Holden *et al.*, 2004). From a clinical perspective, *B. pseudomallei* is intrinsically resistant to most antibiotics and melioidosis is difficult to diagnose, particularly in non-endemic regions where selective culture media are not routinely used. The risk of contracting melioidosis is heightened in certain individuals, particularly those with diabetes or impaired immune function. Melioidosis can cause rapid death from septic shock if left untreated or if appropriate antibiotic therapy is delayed (Wiersinga *et al.*, 2012). *B. pseudomallei* is currently classified as a Tier 1 Select Agent in the United States due to the potential for use as a bioweapon, high mortality rate, lack of vaccine and an inhalational route of exposure.

Worldwide, both the recognized distribution of melioidosis and its incidence are increasing. *B. pseudomallei* has now been observed in most tropical and subtropical regions across the globe (Currie, 2015; Dance, 2015). The highest rates of melioidosis are reported in northern Australia and Thailand where *B. pseudomallei* is common in soil and freshwater (Cheng & Currie, 2005; Wiersinga *et al.*, 2012). However, there have been several puzzling, ostensibly autochthonous (i.e. indigenous) outbreaks that have occurred far outside endemic latitudes (20° N and 20° S) (Cheng & Currie, 2005; Yip *et al.*, 2015). These outbreaks, although uncommon, may last for several years, with *B. pseudomallei* frequently recoverable from soil or water in these regions during the outbreak. Perhaps the most notorious and devastating melioidosis outbreak in a non-endemic region occurred in France between 1975 and 1979. Originating in a Parisian zoo, this outbreak was attributed to an infected panda from China, and resulted in the death or slaughter of most of the zoo's animals. There was subsequent widespread infection of horses throughout France and several human melioidosis cases (Mollaret, 1988). Such outbreaks show that *B. pseudomallei* can cause long-term contamination of animal housing, pastures and the wider environment, even in temperate regions. Although *B. pseudomallei* does not form spores, it can survive and persist in a wide range of environments, including nutrient-poor conditions, for years and even decades (Moore *et al.*, 2008; Wuthiekanun *et al.*, 1995).

The advent of bacterial genomics, driven in recent years by inexpensive, accessible and high-throughput next-generation whole-genome sequencing (WGS) technology, has revolutionized microbiology. Applications of WGS in public health, especially for epidemiological and outbreak source tracing investigations, are at the forefront of this transformation (Köser *et al.*, 2012a). The utility of WGS has been demonstrated in hospital and community outbreaks caused by common pathogens such as methicillin-resistant *Staphylococcus aureus* (Köser *et al.*, 2012b), *Klebsiella pneumoniae* (Snitkin *et al.*, 2012), *Vibrio cholerae* (Hendriksen *et al.*, 2011) and *Mycobacterium tuberculosis* (Gardy *et al.*, 2011; Walker *et al.*, 2013), both retrospectively and in real time. WGS is a powerful tool for epidemiological and evolutionary analyses of highly recombinogenic species such as *B. pseudomallei*. Given that melioidosis cases are almost always caused by infection acquired from the environment rather than person-to-person transmission, the data generated from WGS can provide robust detection of phylogeographical signal. Importantly, WGS overcomes the problems of homoplasy and insufficient resolution that plague conventional lower-resolution genotyping methods such as PFGE or MLST.

In the current study, we used WGS to investigate the origin, dissemination and persistence of a quarter-century *B. pseudomallei* outbreak that occurred in the Avon Valley region in south-west Western Australia, from 1966 to 1991 (Currie *et al.*, 1994; Golledge *et al.*, 1992; Ketterer & Bamford, 1967). The Avon Valley is located at 31° S, well outside the conventional latitudes of *B. pseudomallei* endemicity. This infection cluster involved melioidosis cases in multiple animals including sheep, goats, horses and a dog from four farms: two neighbouring properties near Chittering, and two neighbouring properties near Gidgegannup (Ketterer & Bamford, 1967; Lloyd *et al.*, 1988). There was also one human case (Golledge *et al.*, 1992). Over the course of the outbreak, isolates were recovered from soil sampling of one farm in each area. Re-analysis of this outbreak using modern high-resolution WGS provides a unique opportunity to revisit the persistence and molecular evolution of *B. pseudomallei* over a defined timescale in a presumed non-endemic region. Here, we show for the first time an unexpected level of genetic diversity among the Western Australian outbreak isolates, and no clear temporal or geographical pattern of evolution or dissemination. Both these factors suggest low-prevalence endemicity of *B. pseudomallei* in this region, with this bacterium probably persisting longer in this region than previously appreciated. We also show that the *B. pseudomallei* introduced into this region were probably Australian in origin, although the precise origin within Australia could not be determined.

## Methods

### Isolates and DNA extraction.

**Fig. 1.**
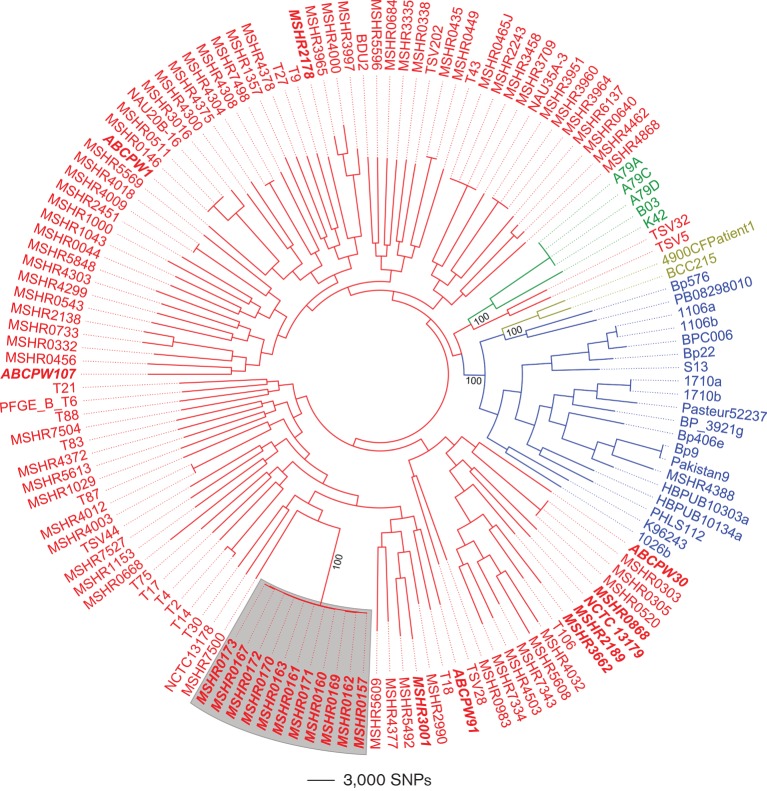
Midpoint-rooted maximum-parsimony phylogeny of 141 global *B. pseudomallei* isolates. This phylogeny was reconstructed using 199 513 orthologous core-genome SNPs identified across all publicly available whole-genome sequences available on GenBank; the 11 Avon Valley outbreak isolates (grey shading) were included for comparison with this global dataset. Red, Australia; blue, Asia; green, Papua New Guinea; gold, South America. Consistency index=0.19. Isolates in bold and italics are from Western Australia; all these isolates were obtained from the endemic region of northern Western Australia, with the exception of NCTC 13179, whose precise origin is not known, and the Avon Valley isolates, which are from south-west Western Australia. Numbers along relevant branches indicate bootstrap support based on 200 replicates.

Eleven *B. pseudomallei* isolates from the 25-year south-west Western Australian outbreak (Currie *et al.*, 1994) were available for our study (Fig. 1). Genomic DNA was extracted from single purified bacterial colonies using the Qiagen DNeasy Blood and Tissue kit as detailed elsewhere (Currie *et al.*, 2007). WGS was performed using the Illumina HiSeq2000 platform.

### Reference genome assembly.

Genome assembly for one of the soil isolates from a Chittering farm (MSHR0169) was performed using mgap (https://github.com/dsarov/MGAP---Microbial-Genome-Assembler-Pipeline), a tool that incorporates Velvet (Zerbino & Birney, 2008), VelvetOptimiser (https://github.com/tseemann/VelvetOptimiser), GapFiller (Boetzer & Pirovano, 2012), abacas (Assefa *et al.,* 2009), image (Tsai *et al.,* 2010), sspace (Boetzer *et al.,* 2011) and icorn2 (Otto *et al.,* 2010). MSHR0169 Illumina reads were aligned against the MSHR0169 assembly using spandx v3.1 (Sarovich & Price, 2014) to manually correct for a small number of spurious variant calls in the assembly. MSHR0169 contigs were reordered against the closed Australian *B. pseudomallei* MSHR1153 genome (GenBank IDs CP009271 and CP009272) using mauve (Darling *et al.,* 2004). The final assembly for MSHR0169 is in 36 high-quality contigs totalling 7 241 312 bp.

### Comparative genomic analysis.

The MSHR0169 assembly was used as the reference genome for comparative genomic analysis of all Avon Valley outbreak isolates using spandx. All SNP and insertion-deletion (indel) variants were visualized in Tablet v1.13.12.17 (Milne *et al.*, 2013) to confirm their accuracy. Genetic loss or addition among outbreak isolates was assessed using the BEDcov output generated by spandx, with MSHR0169 reads used to exclude imperfectly mapped (typically paralogous) regions.

### Phylogenetic analysis.

Our input datasets comprised either a matrix of orthologous, core-genome SNPs across all genomes, or a matrix combining SNPs and orthologous core-genome indels in a binary format to indicate identity compared to the MSHR0169 reference sequence. The latter dataset was included as we have found that, for highly related genomes, combined SNP–indel phylogenetic trees provide a more robust epidemiological signal and greater resolution than SNP-based trees alone (McRobb *et al.*, 2015). Phylogenetic reconstruction was performed using the maximum-parsimony criterion executed in paup v4.0a142 (Swofford, 2002).

To determine the probable phylogeographical origin of the outbreak isolates, we also performed phylogenetic reconstruction of SNPs identified across a global set of *B. pseudomallei* genomes using the maximum-parsimony criterion executed in paup. Genomic data were downloaded from GenBank in FASTA format and converted to synthetic Illumina reads using art vVanillaIceCream (Huang *et al.,* 2012) using quality shift values of 10 prior to analysis with spandx. Contigs <1 kb in length were excluded.

We used 1000 replicates for bootstrapping of all trees except for the global phylogeny, where 200 replicates were used. Trees were visualized using FigTree version 1.4.0 (http://tree.bio.ed.ac.uk/software/figtree/).

### MLST.

MLST was performed on a subset of samples (MSHRs 0157, 0161, 0169, 0170, 0173) using the conventional PCR-based method (Godoy *et al.*, 2003). We also performed MLST *in silico* from assembled WGS data for each isolate, including those previously typed using the traditional PCR method, to confirm sequence types (STs). MLST profiles were deposited in the global *B. pseudomallei* MLST database (http://pubmlst.org/bpseudomallei/).

### Identification of recombination signal.

Identification of SNPs in recombined regions was assessed using Gubbins v1.4.1 (Croucher *et al.,* 2015).

## Results

MLST, both PCR-based and *in silico*, showed that all Avon Valley outbreak isolates belonged to a single ST, ST-284. ST-284 has not been identified outside this outbreak, nor have single-locus variants of ST-284 been documented. Using WGS and maximum-parsimony reconstruction, we compared the ST-284 outbreak isolates with global *B. pseudomallei* reference genomes. Reference sequences were available from several melioidosis-endemic regions around the world, including from northern Western Australia. The Avon Valley strains were closely related to each other, forming a well-supported clade within the global phylogeny (Fig. 1). As has been previously documented (De Smet *et al.*, 2015; Pearson *et al.*, 2009; Price *et al.*, 2016; Sarovich *et al.*, 2016), Australian *B. pseudomallei* isolates form a well-supported clade that is distinct from isolates of non-Australian origin. The Avon Valley strains are situated within this Australian clade. Bootstrap values for terminal and very deep (intercontinental) nodes in the global phylogeny are well supported, consistent with severely restricted gene flow between *B. pseudomallei* populations on different continents, which minimizes homoplasy. In contrast, nodes at intermediate depth are very poorly resolved. The lack of resolution at these nodes is probably due to the high rate of lateral gene transfer among these *B. pseudomallei* populations (Pearson *et al.*, 2009).

**Fig. 2.**
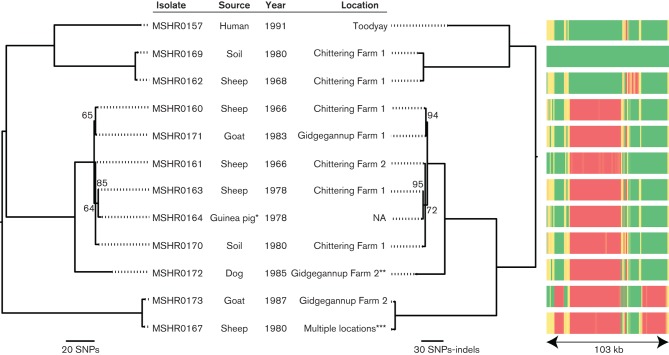
Midpoint-rooted maximum-parsimony phylogenetic reconstruction of Avon Valley isolates based on orthologous core-genome SNPs (left tree; consistency index=0.99) or combined orthologous core-genome SNPs and indels (right tree; consistency index=0.95). Numbers along branches indicate bootstrap support based on 1000 replicates; only bootstrap values <100 are shown. Note the similar topologies but superior bootstrap values for the combined SNP–indel tree. BEDcov locus presence–absence across 1 kb windows, as represented by a heatmap (red, no coverage; green, full coverage), matches the phylogenetic analyses. *MSHR0164, an isolate from a guinea pig infected with MSHR0163, differed from MSHR0163 by two SNPs. **The dog, which lived on Gidgegannup Farm 3, had recently consumed the partially incinerated remains of a goat that died of melioidosis. ***The sheep from which isolate MSHR0167 was retrieved had been moved several times, including spending 6 weeks on Gidgegannup Farm 2. Note that Chittering Farms 1 and 2 are neighbouring farms, as are Gidgegannup Farms 1 and 2.

To gain the highest possible resolution among the Avon Valley isolates, we also reconstructed phylogenies of just the ST-284 isolates based on orthologous core-genome SNPs and on combined data from orthologous SNPs and indels (Fig. 2). The two phylogenies showed similar topology and recovered three well-supported clades; these phylogenetic clades were also supported by patterns of locus presence–absence (Fig. 2). Surprisingly, isolates did not completely cluster together based on the year of isolation, location or source. For example, despite a 17-year collection date difference between MSHR0160 and MSHR0171, which were isolated from a sheep on Chittering Farm 1 and a goat on Gidgegannup Farm 1, respectively, these isolates differed by just one SNP (Fig. 2). In contrast, MSHR0169 and MSHR0170, which were both collected in 1980 from soil on Chittering Farm 1, differed by 163 SNPs and 109 indels. Of note, strains from the beginning of the outbreak were also genetically distinct. MSHR0160 and MSHR0161, both collected from sheep in 1966 on the neighbouring Chittering Farms 1 and 2, differed by only three SNPs and four indels. In contrast, MSHR0162 was collected from a sheep on Chittering Farm 1 only 2 years later, yet it had 268 differences from MSHR0160 (161 SNPs and 107 indels) and 267 differences from MSHR0161 (160 SNPs and 107 indels).

**Fig. 3.**
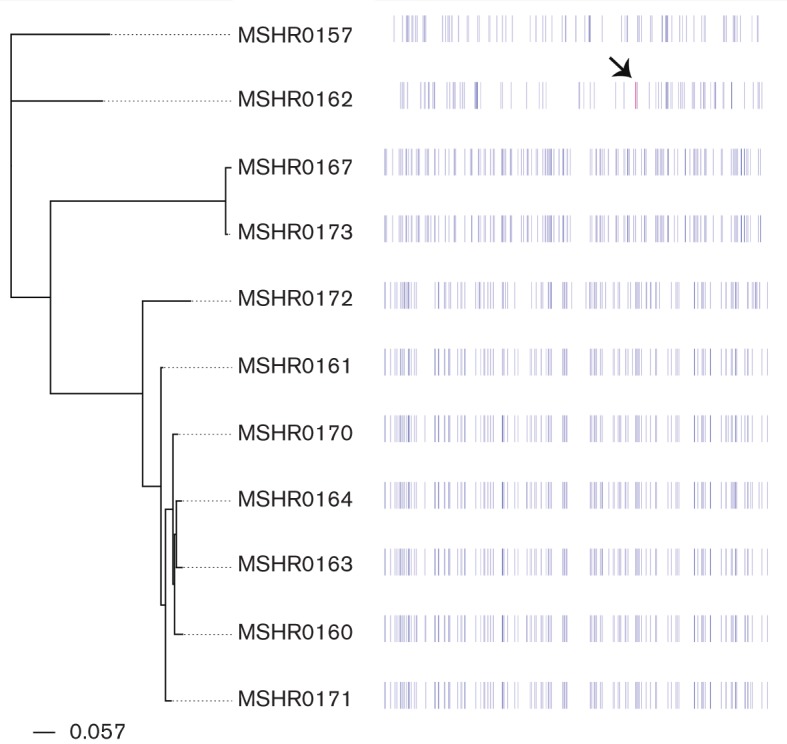
Gubbins analysis of the Avon Valley *B. pseudomallei* genomes. Blue lines indicate SNPs; the magenta line (indicated by a black arrow) in MSHR0162 denotes a predicted block of 25 recombinogenic SNPs within a 14.4 kb region.

To determine the relative contribution of recombination to mutation in generating genetic diversity in the Avon Valley isolates, the distribution of SNPs was examined using Gubbins. This analysis did not find extensive evidence of recombination among the outbreak strains, with a single 14.4 kb locus containing 25 potentially recombinogenic SNPs observed in MSHR0162; no recombinogenic SNPs were identified in the other strains (Fig. 3).

## Discussion

Globally, most melioidosis cases occur in tropical and subtropical regions where *B. pseudomallei* is endemic, although evidence is mounting that this bacterium can persist in cooler and more arid locales. In our study, we used WGS to investigate an historical melioidosis outbreak in the temperate Avon Valley region, Western Australia, which spanned a 25-year period between 1966 and 1991. It has previously been speculated that this outbreak began following the introduction of an infected animal from an endemic region in northern Australia (Golledge *et al.*, 1992; Ketterer & Bamford, 1967). Ketterer & Bamford (1967) described two horses that were imported to the Avon Valley in 1965 from the Kimberley region in northern Western Australia, where melioidosis is known to be endemic. Both horses died of a disease, confirmed to be melioidosis at autopsy. Unfortunately, no isolates from these horses were kept. Subsequent studies using low-resolution ribotyping and RAPD analyses (Currie *et al.*, 1994; Haase *et al.*, 1995) on the retrieved outbreak isolates suggested that the outbreak was clonal, consistent with a single introduction hypothesis. Likewise, we found in the present study that all the outbreak isolates shared the same MLST genotype, ST-284. However, these methods all suffer from relatively low resolution, as they only report differences at a limited number of genetic loci, and are therefore insufficient for revealing the probable origin and evolutionary patterns of this outbreak.

To combat the issues faced with lower-resolution methods, we performed WGS on 11 available *B. pseudomallei* isolates from the Avon Valley outbreak, plus an additional isolate obtained from a guinea pig infected with MSHR0163. Comparison of these *B. pseudomallei* genomes revealed unexpected and previously unobserved genetic diversity, with SNP and indel variation already present among isolates considered to represent the beginning of the outbreak. For example, 268 genetic differences were found between MSHR0160 and MSHR0162; these strains were isolated in 1966 and 1968, respectively, from sheep on Chittering Farm 1. This result is consistent with previous genotyping completed on isolates using 30-locus multilocus variable number tandem repeat analysis, which found all the outbreak isolates had unique, yet closely related genotypes (Pearson *et al.*, 2007).

The *B. pseudomallei* genome is highly recombinogenic, and substantial genetic diversity can arise quickly via recombination with other strains. For example, we recently identified a large, ~1.3 Mbp recombination event in a clonal *B. pseudomallei* population from an Australian melioidosis patient; indeed, this single recombination event accounted for at least 94 % of the genetic variation among these clonal strains (Price *et al.*, 2015). In contrast, our bioinformatic analysis of the Avon Valley outbreak isolate genomes failed to identify a strong signal of recombination (Fig. 3). Given the propensity for *B. pseudomallei* to recombine, it cannot be ruled out that recombination with a closely related clone has led to some of the observed variation. However, we believe that recombination is unlikely to have contributed to the genetic diversity seen among the Avon Valley outbreak isolates. Firstly, no other *B. pseudomallei* ST has ever been found in this region, suggesting that few or no other *B. pseudomallei* strains exist in the environment to drive genetic diversification via recombination. Secondly, SNPs were randomly distributed throughout the ST-284 genome (Fig. 3) rather than being clustered at distinct loci, the latter of which would be expected if recombination with a different ST was responsible for generating genetic diversity (Price *et al.*, 2015). Thirdly, the genetic diversity of these outbreak isolates is greater than could have evolved *in situ* by point mutation in the Avon Valley if a putative single introduction of the bacterium in 1965 is assumed. Based on our collective analysis, we therefore speculate that *B. pseudomallei* has been present in the soil and water of this region for several years, decades or even centuries before the first cases of melioidosis were diagnosed in 1966.

The observed clonality of the Avon Valley isolates using low-resolution typing methods has meant that melioidosis cases in this region have long been characterized as part of an 'outbreak'. However, given the genetic diversity among these strains and the long-term persistence of the bacterium in this region, we propose that these cases should instead be considered as low-prevalence *B. pseudomallei* endemicity. Similar cases of melioidosis recurring across several decades in particular locations outside recognized endemic tropical regions have been documented elsewhere in Australia, notably in South East Queensland and Central Australia. Cases in these regions generally arise following unusual and extreme weather events. For example, following heavy rainfall associated with extensive flooding in 2011, there were six reported autochthonous melioidosis cases in communities near Alice Springs, a normally arid Central Australian desert region (23.8° S) (Yip *et al.*, 2015). Isolates from these cases were unique STs not previously found elsewhere. In addition, environmental samples from this region were *B. pseudomallei*-positive, pointing to the probable endemicity of this organism, even in harsh, desert-like environments (Yip *et al.*, 2015). This pattern of strain diversity is the same as found in cases that have arisen in South East Queensland (27.5° S) following high rainfall and flooding (Munckhof *et al.*, 2001). In contrast, a new region of endemicity has been uncovered recently in Puerto Rico (Doker *et al.*, 2015), in which four cases across 14 years showed the same ST (or a single-locus variant of that ST), a similar pattern to that seen in the Avon Valley cluster.

Tracing the origin of ST-284 introduction into temperate Western Australia is important for understanding the risk posed by the possible spread of *B. pseudomallei* into other temperate regions. Our WGS approach suggests that there may have been a single introduction of *B. pseudomallei* to the region via an infected animal, with subsequent persistence in the soil since then. However, given the genetic diversity of strains, such an introduction probably occurred much earlier than the previously proposed introduction date of ~1965. Recent research has found that polyclonal infections, such as those with multiple strains of the same ST, probably occur more often than has been recognized (Price *et al.*, 2015). Thus, another hypothesis for the origin of the Avon Valley isolates is the more recent introduction of genetically diverse ST-284 strains, such as via an animal with a polyclonal ST-284 infection. ST-284 is clearly of Australian origin (Fig. 2) although we were unable to resolve its precise origin due to inherently poor bootstrap support among strains from Australia (data not shown), even with WGS data. Interestingly, ST-284 has not been found elsewhere, nor have any single-locus variants (SLVs) of ST-284 yet been discovered, suggesting that the geographical origin of this ST has not yet been sampled.

Much remains unknown about the ecology, reproduction and evolution of *B. pseudomallei*, although our study has provided new insights. The bacterium’s propensity for lateral gene transfer complicates attempts to understand phylogenetic relationships within this species. Unlike more clonal species, it is very difficult to calibrate an accurate molecular clock for *B. pseudomallei*. Some previous studies have attempted to address these same issues. For example, serial passaging studies have measured the rate of evolution at variable number tandem repeat loci *in vitro*, and the genetic diversity of isolates from the course of acute and chronic infections demonstrates microevolution in response to selection over defined timescales (Price *et al.*, 2010, 2013). However, while *B. pseudomallei* can mutate rapidly under selection, as has been documented in a 12-day acute infection (Limmathurotsakul *et al.*, 2014; Price *et al.*, 2010), our study shows that this organism can also persist in the environment with very little microevolution over several years and possibly even decades, a finding that has also been recently mirrored in *Francisella tularensis* (Johansson *et al.*, 2014). One explanation for this observation is the possibility for strain mix-ups among these historical isolates, although we deem this unlikely given that there were still no discernible temporal or geographical patterns even when we assumed this scenario. Furthermore, repeat WGS analysis of three original isolate stocks yielded essentially identical genotypes (results not shown). Another possibility is that the bacterium can enter a quiescent, non-replicative state under certain conditions (Inglis & Sagripanti, 2006), a trait that may explain why *B. pseudomallei* can persist so long in ostensibly unfavourable environments. The current uncertainty regarding generation times under different conditions thus limits our ability to determine how long *B. pseudomallei* has been present in temperate Western Australia, and therefore to distinguish between scenarios such as recent polyclonal versus historical monoclonal introduction.

In considering the scenario of an historical introduction, it is significant that the Avon Valley outbreak occurred in one of the earliest sites for sheep farming in Western Australia. Sheep arrived with the European settlers in the region in 1831. Given the protean nature of melioidosis symptoms, it is entirely possible that sheep in this region have been contracting melioidosis for longer than has been recognized. As with human disease, ovine melioidosis causes non-specific symptoms, and indeed has only been recognized in Australia since the 1950s (Cottew *et al.*, 1952). The loss of sheep from unknown causes in south-west Australian flocks during the 19th century was extremely common. For example, an unknown disease killed a number of sheep in a flock on the Swan River (site of modern day Perth) in 1834, causing rapid death with respiratory distress and neurological symptoms, and post-mortem evidence of encephalitis (O'Brien, 1834). Deaths in the flock stopped when animals were removed to higher, drier ground. It was thought to be a disease known as 'sheep catarrh', which was then ravaging flocks in New South Wales, and from which the Swan River colony obtained its stock. The cause of the 'sheep catarrh' epidemic remains unknown, but melioidosis in travelling flocks from endemic regions is one possibility consistent with many of the symptoms and epidemiological characteristics of the disease.

Endemic *B. pseudomallei* in temperate Western Australia has a number of important public health implications. Firstly, grazing animals are especially susceptible to melioidosis, particularly sheep and goats (Choy *et al.*, 2000), which may increase the risk of zoonotic transmission to immunocompromised individuals with melioidosis risk factors. Although very uncommon, zoonotic transmission to humans has been observed, with three probable Australian cases documented (Choy *et al.*, 2000). Those in close contact with susceptible agricultural animals, such as farmers, or those who consume unpasteurized milk or meat from such animals may be at higher risk. The high potential inoculum present in infected sheep or goats may also increase the risk of transmission to other less susceptible animals. An instance of transmission between two animals is likely to have occurred in the Avon Valley, as the dog that contracted melioidosis at Gidgegannup Farm 2 died within a few weeks of consuming the partially incinerated remains of an infected goat (Lloyd *et al.*, 1988). Although *B. pseudomallei*-negative results were obtained from soil sampling efforts at Gidgegannup Farm 2 in 1999 (N. Buller, pers. comm.), 8 years after the last documented melioidosis case in the Avon Valley region, the ongoing presence of *B. pseudomallei* in this region cannot be definitively ruled out. Unusually high rainfall or flooding in Avon Valley may unmask further cases in the region, potentially when immunocompromised individuals are exposed to the pathogen via waterlogged soil or floodwater, as has occurred in temperate Queensland and Central Australia (Munckhof *et al.*, 2001; Scott *et al.*, 1997; Yip *et al.*, 2015).

In conclusion, we have used WGS to characterize genome-wide changes occurring in environmental, animal and human *B. pseudomallei* isolates from a 25-year melioidosis outbreak in temperate Australia. All isolates shared the same ST and were highly related. We observed genetic diversity among the isolates at the whole-genome level but little evidence of recombination. We also observed patterns of mutation that did not correlate with time, geographical region or host species. Higher than expected genetic diversity was present among isolates collected in the 1960s, when melioidosis was first recognized in this region, a finding that challenges the convention that temporal and geographical distance correlate with evolutionary relatedness. These results suggest that *B. pseudomallei* has been present in the temperate Avon Valley region of Western Australia for much longer than has been previously recognized, and we propose that the Avon Valley may in fact be a region of low-prevalence *B. pseudomallei* endemicity. Despite the last reported case being in 1991, we anticipate that further cases may arise if conditions favourable for *B. pseudomallei* occur. Our study provides further compelling evidence of the long-term environmental persistence of *B. pseudomallei* outside traditional endemic regions, with implications that extend to other temperate zones across the globe. Finally, although a recent, comprehensive modelling study predicted far greater *B. pseudomallei* global distribution and incidence than has been previously recognized (Limmathurotsakul *et al.*, 2016), our results show that future modelling efforts need also to consider the possibility of low-prevalence *B. pseudomallei* endemicity in temperate regions.
